# Cortical strut allograft reconstruction for aseptic loosening of knee arthrodesis with a severe femoral defect: a case report

**DOI:** 10.1093/jscr/rjag488

**Published:** 2026-06-25

**Authors:** Joao Pinheiro, Tobias Baumgärtner, Alexander Blümke, Mohamad Bdeir, Sascha Gravius, Ali Darwich

**Affiliations:** Orthopaedic and Trauma Surgery Center, Mannheim University Hospital, Heidelberg University, Theodor-Kutzer-Ufer 1-3, 68167 Mannheim, Germany; Orthopaedic and Trauma Surgery Center, Mannheim University Hospital, Heidelberg University, Theodor-Kutzer-Ufer 1-3, 68167 Mannheim, Germany; Orthopaedic and Trauma Surgery Center, Mannheim University Hospital, Heidelberg University, Theodor-Kutzer-Ufer 1-3, 68167 Mannheim, Germany; Orthopaedic and Trauma Surgery Center, Mannheim University Hospital, Heidelberg University, Theodor-Kutzer-Ufer 1-3, 68167 Mannheim, Germany; Orthopaedic and Trauma Surgery Center, Mannheim University Hospital, Heidelberg University, Theodor-Kutzer-Ufer 1-3, 68167 Mannheim, Germany; Orthopaedic and Trauma Surgery Center, Mannheim University Hospital, Heidelberg University, Theodor-Kutzer-Ufer 1-3, 68167 Mannheim, Germany

**Keywords:** knee arthrodesis, aseptic loosening, revision surgery, cortical strut allograft, femoral bone loss, cerclage cable fixation

## Abstract

Knee arthrodesis is a rare but established salvage procedure after periprosthetic joint infection or mechanical failure. We report the case of a 74-year-old woman with aseptic loosening of a knee arthrodesis and a Paprosky type IV femoral defect. Reconstruction with a cortical strut allograft and modular arthrodesis revision resulted in an uncomplicated postoperative course and marked functional improvement. This approach may represent a viable alternative for revision in complex femoral defect situations.

## Introduction

Knee arthrodesis is an established salvage procedure after failed total knee arthroplasty, most frequently in the setting of periprosthetic joint infection [1]. Despite high fusion rates across different techniques, complication rates remain substantial, with mechanical failure and reinfection representing the major causes of revision [1–3]. Revision becomes particularly challenging in the presence of extensive femoral bone loss, as additional resection may be required, which can compromise stem fixation and necessitate alternatives such as total femur replacement, thereby increasing morbidity and complication rates [4]. Cortical strut allografts are a well-known method for restoring structural bone support in complex reconstructions [5]. Their role in revising a loosened knee arthrodesis with severe femoral defects is rarely reported. We present a case of aseptic loosening of a knee arthrodesis with a Paprosky type IV femoral defect treated with cortical strut allograft reconstruction and modular arthrodesis revision.

## Case report

A 74-year-old woman presented with progressive right thigh pain for over a year without trauma and loss of ambulation, resulting in wheelchair dependence. She had undergone right knee arthrodesis in 2011 following periprosthetic joint infection after primary total knee arthroplasty with multiple revision procedures. She reported no fever or systemic infectious symptoms.

On examination, the scar was dry without erythema or increased warmth. Tenderness was present from the distal femur to the knee region. Distal neurovascular status was intact. Laboratory testing showed no evidence of active infection, with normal erythrocyte sedimentation rate (ESR), C-reactive protein (CRP), and leukocyte count.

**Figure 1:**
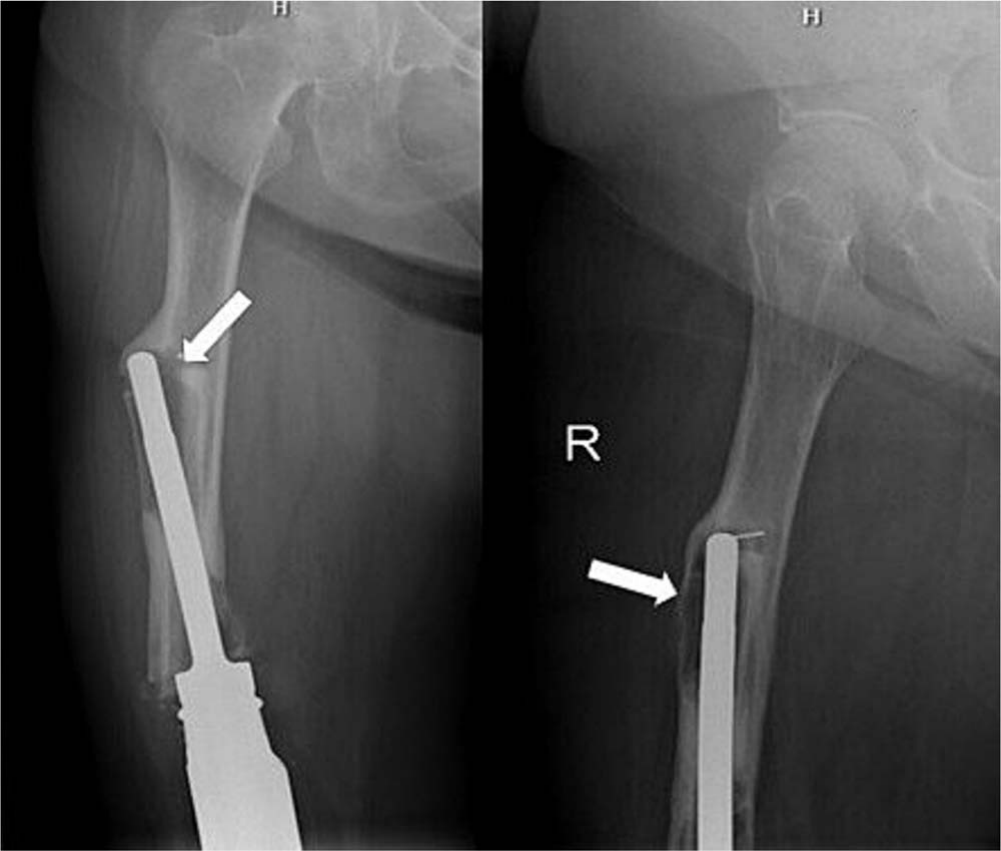
Two-view radiographs of the femur demonstrating radiographic signs of loosening of the cemented intramedullary stem component.

Plain radiographs revealed loosening of the femoral arthrodesis component with osseous overgrowth, consistent with chronic loosening (Fig. 1). Computed tomography of the entire limb confirmed loosening of the femoral component. In addition, lateral cortical perforation and extensive osteolysis with severe bone loss consistent with a Paprosky type IV defect were observed. The tibial component appeared well fixed without radiographic signs of loosening.

**Figure 2:**
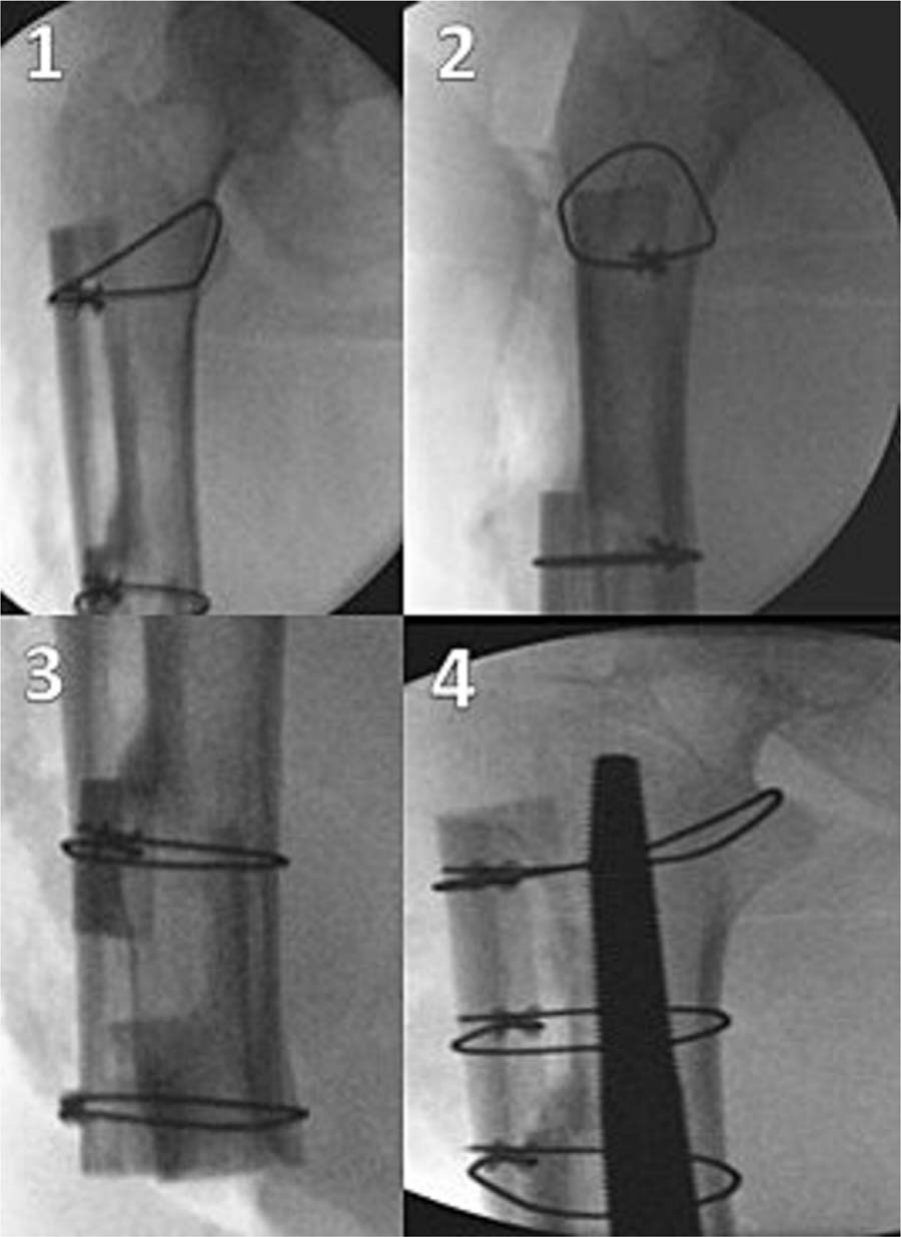
Intraoperative reconstruction. Panels 1–3 show correct positioning of the cortical strut graft secured with cerclage cables. Panel 4 shows the cementless femoral component in situ.

A one-stage revision of the femoral component was performed for aseptic loosening. After explantation of the cemented femoral component, the cement plug and cement mantle, femoral reconstruction was achieved using a split allogeneic femoral diaphyseal cortical strut graft (250 mm; German Institute for Cell and Tissue Replacement, DIZG) secured with cable cerclages. A cementless femoral component (Mutars RS stem 15 × 200 mm, Implantcast) was implanted, and the arthrodesis module was exchanged and aligned at 20° external rotation (Fig. 2). Planned limb shortening was 1.5 cm. Multiple tissue samples were obtained for microbiological and histological analysis.

**Figure 3:**
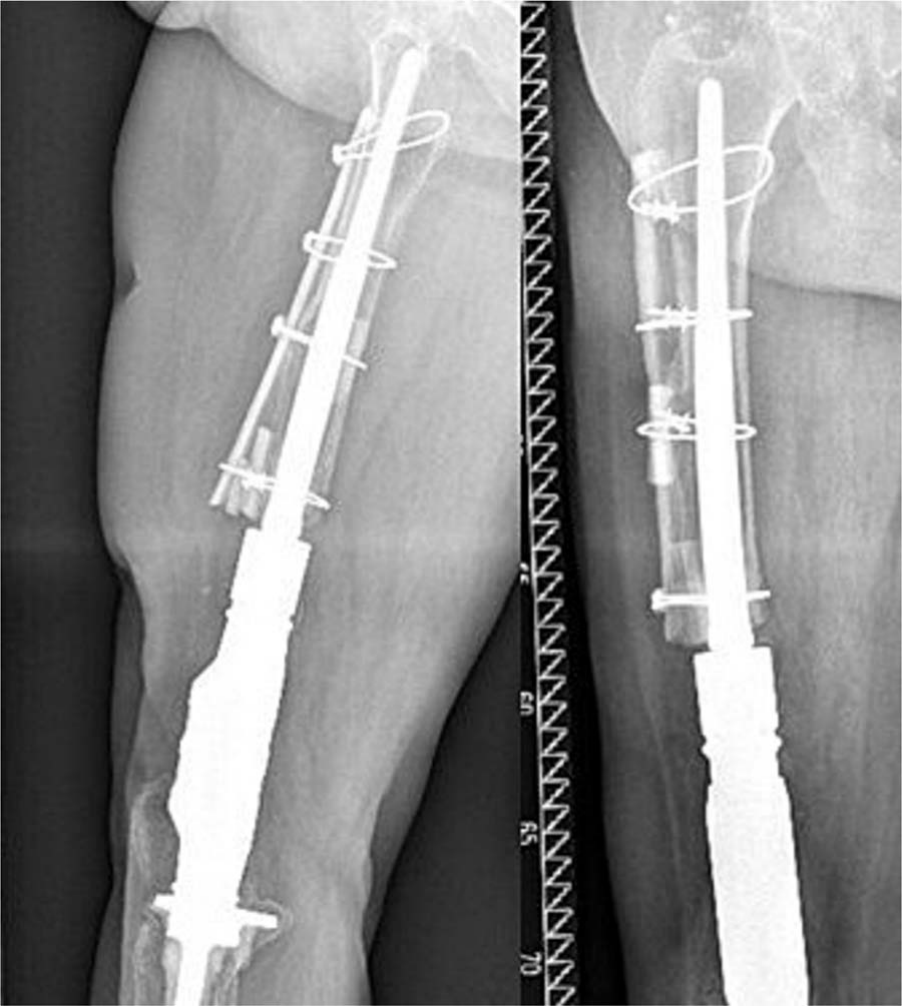
Postoperative two-view radiographs following reconstruction with a cortical strut graft and modular knee arthrodesis.

The postoperative course was uncomplicated with primary wound healing. Mobilization was initiated with partial weight bearing (20 kg) for 6 weeks. Postoperative radiographs confirmed correct implant position (Fig. 3). Microbiological and histological analysis of the samples showed no signs of acute periprosthetic infection. Follow-up at 1, 6, and 12 months included clinical and radiographic assessment.

**Figure 4:**
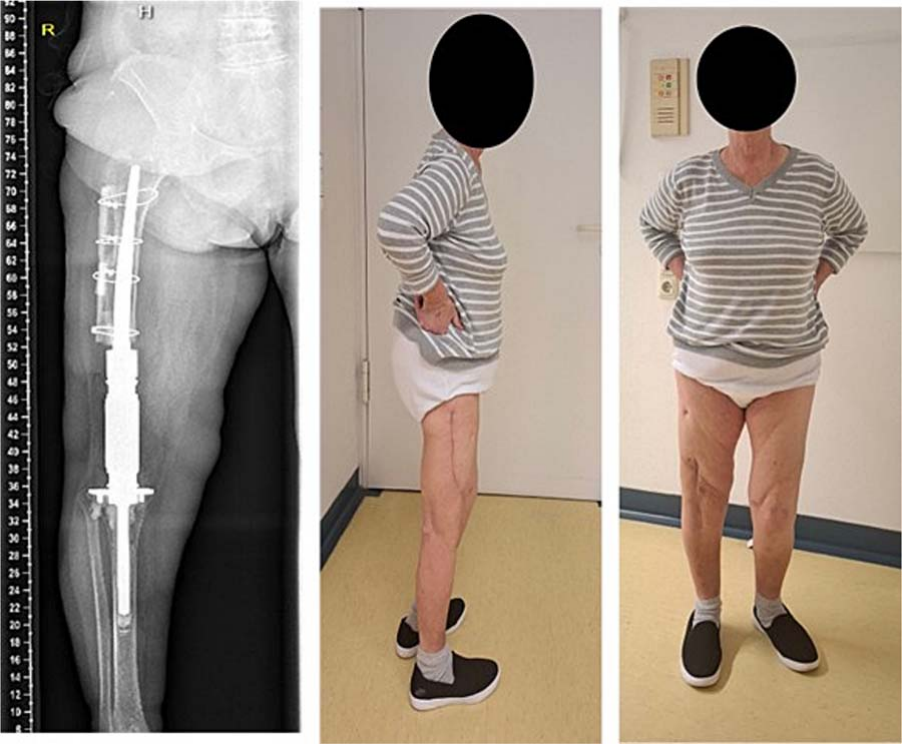
Postoperative outcome demonstrating stable arthrodesis fixation with incorporation of the cortical strut graft. Clinically, a leg length discrepancy of 1.5 cm was present. The wound was dry and non-irritated, and intraoperative samples were negative.

For outcome assessment, VAS, Patient Global Impression of Change (PGIC), EQ-5D-5L Index, Oxford Knee Score (OKS), Western Ontario and McMaster Universities Osteoarthritis index (WOMAC), and SF-36 were recorded preoperatively and during follow-up. At 12 months, pain decreased substantially (VAS 9 → 3), and she rated her overall status as ‘very much improved’ (PGIC 7/7). Overall health status improved (EQ-5D-5L −0.33 → 0.64), most notably in mobility, pain/discomfort and anxiety/depression. Function also improved (OKS 8 → 24; WOMAC 80 → 40). SF-36 domain scores, which had been below 30 in all domains preoperatively, increased postoperatively to a mean of ~48, reflecting gains in physical functioning and pain as well as mental well-being. She denied neuropathic pain; however, a sensory disturbance over the scar persisted. Serial radiographs demonstrated stable fixation and progressive incorporation of the cortical strut allograft without signs of loosening (Fig. 4).

## Discussion

Knee arthrodesis remains a salvage option after failed total knee arthroplasty, most commonly after complex revision for periprosthetic joint infection [2, 3]. Despite generally reliable fusion, the procedure carries a substantial complication burden, and mechanical failure and recurrent infection remain leading causes for reoperation [1–3]. Reported overall complication rates average ~30%, with some series describing rates up to 45%, while the risk of mechanical failure ranges from 5% to 15% [6, 7]. In addition, arthrodesis can impose substantial mental and socioeconomic consequences for affected patients [1, 6].

Revision of arthrodesis becomes particularly challenging in the presence of severe femoral bone loss, where the main goals are to re-establish a stable and strong femoral structure, and thus, preserve limb length while avoiding further compromise of remaining host bone. To overcome this, additional femoral resection with limb shortening and use of a longer stem is a common strategy to regain fixation and maintain length [5]. In the present case, limited residual femoral length and a segmental defect made further resection impractical and risked escalation to a more extensive reconstruction. Conversion to total femur replacement would have been an alternative; however, it would represent a major undertaking with increased morbidity and mechanical risk [4].

Cortical strut allograft augmentation can offer both mechanical and biological advantages in this setting: it restores cortical continuity, improves load transfer and resistance to bending, and provides a scaffold for incorporation. In our patient, a split diaphyseal strut graft secured with cerclage cables enabled a segment-preserving reconstruction and supported reimplantation of a cementless modular arthrodesis stem. Given the absence of clinical and laboratory signs of infection preoperatively, a single-stage approach was chosen; this was subsequently supported by negative intraoperative tissue samples. The uncomplicated recovery, radiographic graft incorporation, and clinically meaningful improvement at 12 months suggest that strut allograft augmentation may be considered an option for selected arthrodesis revisions with extensive femoral defects. Further study is needed to define durability beyond short-term follow-up.

### Clinical relevance

Cortical strut allograft augmentation can enable segment-preserving reconstruction and durable fixation in aseptic revision of knee arthrodesis with extensive femoral bone loss.
